# New Flavanol and Cycloartane Glucosides from *Landoltia punctata*

**DOI:** 10.3390/molecules19056623

**Published:** 2014-05-22

**Authors:** Nini Wang, Guobo Xu, Yang Fang, Tao Yang, Hai Zhao, Guoyou Li

**Affiliations:** Key Laboratory of Environmental and Applied Microbiology, Chengdu Institute of Biology, Chinese Academy of Sciences, Chengdu 610041, China

**Keywords:** *Landoltia punctata*, flavanol glucoside, cycloartane glucoside, antioxidant

## Abstract

Chemical investigation on the constituents of *Landoltia punctata* led to the isolation and identification of 17 compounds, four of which were new and identified as (3*β,*24*S*)-9,19-cycloartane-3,22,24,25-tetraol 3-*O*-[*β*-d-glucopyranosyl-(1→2)]-[*β*-d-glucopyranosyl-(1→6)]-*β*-d-glucopyranoside (**1**), (3*β,*24*S*)-9,19-cycloartane-3,24,25-triol 3-*O*-[*β*-d-glucopyranosyl-(1→2)]-[*β*-d-glucopyranosyl-(1→6)]-*β*-d-glucopyranoside (**2**), 3,4'-dihydroxy-7,3'-dimethoxyflavan-5-*O*-*β*-d-glucopyranoside (**3**) and 3,4'-dihydroxy-4,7,3'-trimethoxyflavan-5-*O*-*β*-d-glucopyranoside (**4**). Their structures were elucidated by spectroscopic, chemical, and biochemical methods. Thus, cycloartane triterpenoids were discovered in the Lemnaceae family for the first time. Compound **3** showed antioxidant capacity in the positively charged 2,2'-azino-bis-3-ethylbenzothiazoline-6-sulfonic acid radical (ABTS^+•^) and superoxide anion radical scavenging assays.

## 1. Introduction

The plant of the Lemnaceae, commonly known as duckweed, is a kind of aquatic monocot angiosperm and the smallest with flowers, which is widely distributed on the surface of still, slowly flowing, and polluted waters around the World. In this family, there are 37 species, representing five genera: *Lemna*, *Landoltia*, *Spirodela*, *Wolffia* and *Wolffiella* [1,2]. Duckweeds grow very fast and proliferate quickly by budding, allowing them to colonize freshwater habitats rapidly and produce 13 to 38 metric tons/hectare/year dry weight of plant mass [3,4]. Since duckweeds can absorb pollutants (eg. N, P and heavy metals) from wastewater, they have been commonly used in the treatment of domestic and animal wastewater streams for many years [5,6,7]. Meanwhile, because of the relatively high starch and low lignin content in this plant, it has been proved to be an ideal bioresource for bioethanol production [4,8]. Supported by the Minister of Science and Technology and the Major Projects of Knowledge Innovation Program of Chinese Academy of Sciences, our group have been focused on the exploitation of duckweed in the fields of biofuels and natural products. 

In our previous study, *Landoltia* *punctata* demonstrated obvious advantages in the biofuel aspect for its higher starch content and easier acquisition among the five genera in Lemnaceae [9]. Large scale cultivation of *L. punctata* has been carried out by our group, and tons of *L. punctata* biomass can be obtained in a very short time. It is no doubt that *L. punctata* has become a new resource for natural products such as proteins, polysaccharides, amino acids, and other small molecules. 

**Figure 1:**
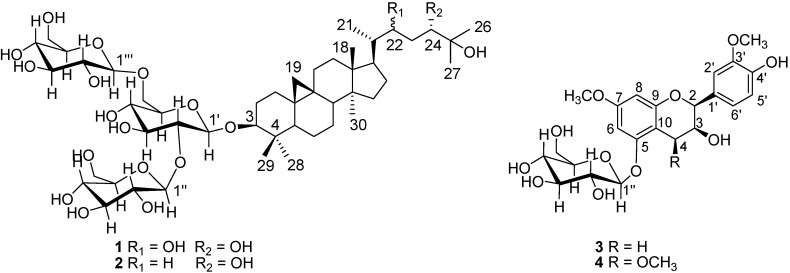
Chemical structures of **1**–**4**.

There are a few reports about the chemical composition of *L. punctata*. To date, only eight compounds have been reported. One is an anthocyanin, and the other seven isolated by paper chromatography were determined by R_f _values, UV spectral maxima and color reactions to be saponarin, isosaponarin, saponaretin and the glucosides of saponaretin and vitexin [10]. Detailed chemical exploration on this plant is imperative. In this study, we investigated the chemical components of *L. punctata* by chromatographic and spectroscopic methods. As a result, four new compounds: (3*β,*24*S*)-9,19-cycloartane-3,22,24,25-tetraol 3-*O*-[*β*-d-glucopyranosyl-(1→2)]-[*β*-d-glucopyranosyl-(1→6)]-*β*-d-glucopyranoside (**1**), (3*β,*24*S*)-9,19-cycloartane-3,24,25-triol 3-*O*-[*β*-d-glucopyranosyl-(1→2)]-[*β*-d-glucopyranosyl-(1→6)]-*β*-d-glucopyranoside (**2**), 3,4'-dihydroxy-7,3'-dimethoxyflavan-5-*O*-*β*-d-glucopyranoside (**3**) and 3,4'-dihydroxy-4,7,3'-trimethoxyflavan-5-*O*-*β*-d-glucopyranoside (**4**), (Figure 1), together with 13 known ones apigenin (**5**), luteolin (**6**), apigenin-7-*O*-*β*-glucoside (**7**), luteolin-7-*O*-*β*-glucoside (**8**) [11], vitexin (**9**), isovitexin (**10**), orientin (**11**), isoorientin (**12**) [12], 6,8-di-*C*-*β*-glucosylapigenin (**13**) [13], 6-*C*-*β*-glucosyl-8-*C*-*β*-galactosylapigenin (**14**) [14], *β*-sitosterol (**15**), stigmasterol (**16**) [15], 5,22-diene-3,6-dicarbonyl-stigmasterol (**17**) [16] were isolated and identified from the 95% ethanol extract of *L. punctata*. 

This is the first report of cycloartane-type triterpenoids from the Lemnaceae family. Here the isolation and structural elucidation of these compounds as well as bioactivities of compound **3** were described.

## 2. Results and Discussion

### 2.1. Structure Elucidation of Compounds **1**–**4**

Compound **1** was obtained as an amorphous powder. Its HR-ESI-MS quasi-molecular ion peak at *m/z* 985.5371 ([M+Na]^+^) corresponded to a molecular formula of C_48_H_82_O_19_, which was supported by the NMR signals. ^1^H-NMR spectrum of **1** showed characteristic signals of a cyclopropane CH_2_ at δ_H_ 0.64 (*d*, *J* = 3.72 Hz, H-19a) and 0.44 (*d*, *J* = 3.72 Hz, H-19b), six tertiary Me groups (δ_H_ 1.11, 1.24, 1.23, 1.13, 0.95, 1.01; respectively, H_3_-18, H_3_-26, H_3_-27, H_3_-28, H_3_-29, H_3_-30) and a secondary Me group at δ_H_ 0.95 (H_3_-21) [17,18]. Additionally, three anomeric proton signals were observed at δ_H_ 4.74 (*d*, *J* = 7.68 Hz), 4.52 (*d*, *J* = 7.20 Hz) and 4.48 (*d*, *J* = 7.74 Hz), indicative of the presence of three *β*-linked sugar units. The ^13^C-NMR and DEPT spectra permitted differentiation of the 48 resonances into 6 C, 22 CH, 13 CH_2_, and 7 CH_3_ groups, of which 30 were attributed to a triterpene skeleton and 18 to three hexose groups. Acid hydrolysis of **1** gave d-glucoses with optical rotation of 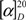
+ 51.2, which was determined by TLC analysis and optical rotation measurement [19,20]. Therefore, **1** was considered to be a cycloartane-type triterpene glucoside. 

**Figure 2:**
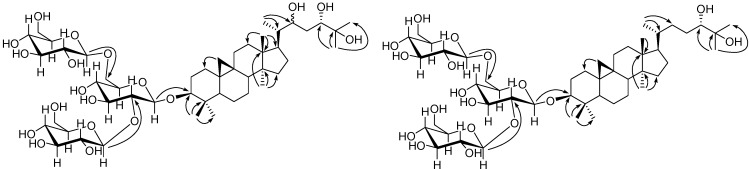
Key HMBC correlations of **1** and **2**.

**Figure 3:**
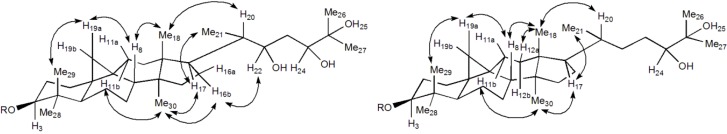
Key NOE correlations of **1** and **2**.

In the HMBC spectrum (Figure 2), correlations of δ_H_ 1.13 (H-28) and δ_H_ 0.95 (H-29) with δ_C_ 90.9 (C-3) suggested that C-3 was substituted by the OH group; 26-CH_3_, 27-CH_3_ with C-24 (δ_C_ 76.2) and C-25 (δ_C_ 74.0), and 21-CH_3_ with C-22 (δ_C_ 71.3) indicated the presence of 24-OH, 25-OH and 22-OH respectively. The glycosidic linkage was determined on the basis of following key spectral signals: HMBC correlations of H-1' with C-3, H-1'' with C-2', and H-1''' with C-6', ^1^H,^1^H-COSY cross peaks of H-1'/H-2', H-6'/H-5', and the NOESY cross peaks of H-1'/H-3' and H-5'. Furthermore, the stereochemistry of C-24 was assigned to be *S* by comparing its spectral data with those reported for analogs [21,22]. Additionally, NOESY correlations between δ_H_ 1.62 (H-8) and δ_H_ 0.44 (H-19a)/18-CH_3_, 18-CH_3_ and δ_H_ 1.79 (H-20), δ_H_ 0.44 (H-19a) and 29-CH_3_, δ_H_ 2.04 (H-16b) and δ_H_ 4.01 (H-22)/30-CH_3_, 21-CH_3_ and δ_H_ 1.80 (H-17), H-17 and 30-CH_3_ were observed, revealing their relative configuration (Figure 3).

Consequently, the structure of **1** was established as (3*β,*24*S*)-9,19-cycloartane-3,22,24,25-tetraol 3-*O*-[*β*-d-glucopyranosyl-(1→2)]-[*β*-d-glucopyranosyl-(1→6)]-*β*-d-glucopyranoside. 

The HR-ESI-MS spectrum of **2** (*m/z* 969.5406 [*M* + Na]^+^, calc. for C_48_H_82_O_18_Na) supported a molecular formula of C_48_H_82_O_18_. The NMR spectra of **2** were very similar to those of **1**, except for one less oxygenated methine at δ_C_ 71.3. Detailed inspection of the HMBC spectrum led to a conclusion that **2** had one less OH on C-22 than **1**, on the basis of the key HMBC correlation of 21-CH_3_ (δ_H_ 1.01) with C-22 (δ_C_ 23.2). The linkage of three glucoses on the triterpenoid aglycon was determined to be the same way as **1** on the basis of HMBC and ^1^H,^1^H-COSY signals. The stereochemistry of **2** was determined on the basis of NOESY cross peaks of H-8 and H-19a/18-CH_3_, 18-CH_3_ and H-12a, H-19a and 29-CH_3_, H-16b and 30-CH_3_, 21-CH_3_ and H-17, H-17 and 30-CH_3_. Acid hydrolysis of **2** gave d-glucoses, which was identified by comparing the optical rotation value with an authentic sample. Finally the structure of **2** was determined to be (3*β,*24*S*)-9,19-cycloartane-3,24,25-triol 3-*O*-[*β*-d-glucopyranosyl-(1→2)]-[*β*-d-glucopyranosyl-(1→6)]-*β*-d-glucopyranoside.

Compound **3** was obtained as colorless needles. Its molecular formula was deduced to be C_23_H_28_O_11 _from the quasi-molecular ion peak at *m/z* 479.1549 ([M*–*H]^−^) in the HR-ESI-MS spectrum, indicating 10 degrees of unsaturation. The IR spectrum revealed the existence of OH (3434 cm^−1^) and aromatic ring (1623 cm^−1^). The ^1^H-NMR spectrum displayed two meta-coupled protons at δ_H_ 6.49 (*d*, *J* = 2.16 Hz) and 6.29 (*d*, *J* = 2.16 Hz), and three ABX system aromatic protons at δ_H _7.22 (*d*, *J* = 1.06 Hz), 6.99 (*dd*, *J* = 8.16, 1.06 Hz), and 6.87 (*d*, *J* = 8.16 Hz). In addition, two methoxyl H-atoms at δ_H_ 3.82 (*s*, 3H) and 3.95 (*s*, 3H), two CH at δ_H _4.29 (br. *s*) and 4.98 (br. *s*), and one CH_2_ at δ_H_ 3.09 (*d*, *J* = 17.60 Hz, 1H) and 3.02 (*dd*, *J* = 17.60, 4.38 Hz, 1H) were observed. Meanwhile an anomeric H-atom at δ_H_ 4.95 (*d*, *J* = 7.20 Hz) together with the signals at δ_H_ 3.42-3.55 indicated the presence of a *β*-linked glycosyl group. Enzymatic hydrolysis of **3** with *β*-d-glucosidase afforded d-glucose, which was identified by direct comparison with the authentic sample by TLC [19]. In view of above evidences, it was concluded that **3** was a flavanol glucoside.

**Figure 4:**
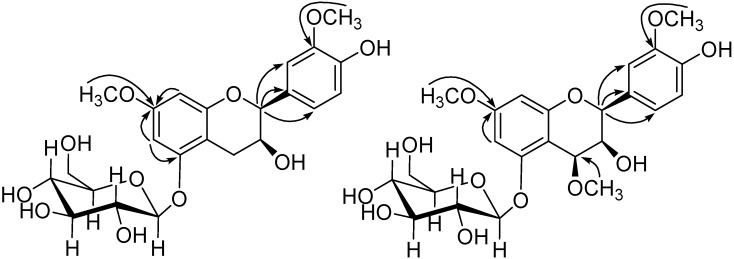
Key HMBC correlations of **3** and **4**.

**Figure 5:**
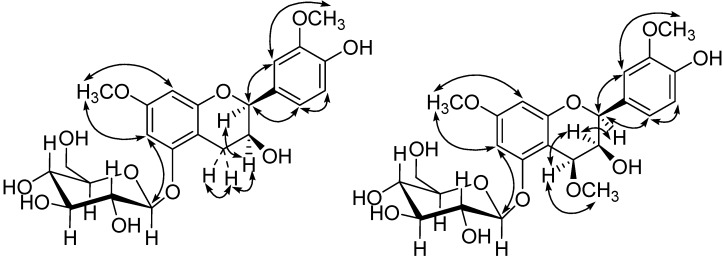
Key NOE correlations of **3** and **4**.

In the combination of the ^13^C-NMR and HSQC spectra of **3**, the 23 carbon resonances could be easily attributed to a flavanol moiety, a glucopyranose unit (δ_C_ 62.7, 71.6, 75.1, 78.2, 78.4, 102.8), and two methoxyls (δ_C _56.0, 56.6). In order to determine the location of substituent groups on the flavanol moiety, HMBC and NOESY experiments were performed. As a result, the NOESY correlations of the methoxyl H-atoms at δ_H _3.82 with H-6 and H-8, H-6 with H-1', and the other methoxyl H-atoms at δ_H _3.95 with H-2', together with the HMBC correlations of 7-OCH_3_ to C-7, 3'-OCH_3_ to C-3', indicated the glucose and two methoxyls were situated at C-5, C-7, and C-3' respectively (Figure 4 and Figure 5).

The NOESY correlation of δ_H_ 4.98 (H-2)/δ_H_ 4.29 (H-3) and resonance of H-2 as a broad singlet indicated that the relative configuration of 2,3 was cis [23,24,25,26,27,28]. Therefore, the structure of **3** was elucidated to be *cis*-3,4'-dihydroxy-7,3'-dimethoxyflavan-5-*O*-*β*-d-glucopyranoside.

Compound **4** was isolated as amorphous powder. The molecular formula was C_24_H_30_O_12_, determined by negative-ion at *m/z* 509.1662 in the HR-ESI-MS. Enzymatic hydrolysis of compound **4** with *β*-d-glucosidase gave d-glucose. Comparing its NMR spectra with those of **3**, it was evident that **4** contained one more methoxyl at δ_H_ 3.65/δ_C_ 56.6 and one oxygenated CH (δ_C_ 73.5) than **3**. 

The location of one more methoxyl at C-4 was determined by the HMBC correlation of 4-OCH_3_ at δ_H_ 3.65 with C-4 (δ_C _73.5). The other substitutions of **4** were confirmed by the same way as **3**.

The configuration of **4** was elucidated on the basis of NOESY correlations and coupling constants. Resonances of H-2 as a broad singlet and H-4 as a small doublet (*J* = 2.28 Hz) were observed, which suggested that the relative 2,3/3,4-stereochemistry were both *cis* [26]. Additionally the NOESY correlation between δ_H_ 5.05 (H-2)/δ_H_ 4.62 (H-4) also supported the above assignment. The structure of **4** was identified to be *cis*-3,4'-dihydroxy-4,7,3'-trimethoxyflavan-5-*O*-*β*-d-glucopyranoside. 

### 2.2. Biological Activity Assay

According to previous bioactivity reports on flavonoids [29,30,31], antimicrobial and antioxidant activities of compound **3** were evaluated. Two pathogenic bacteria (*Bacillus subtilis*, *Escherichia coli*) and two fungi (*Aspergillus niger*, *Saccharomyces cerevisiae*) were selected for antifungal and antibacterial assays, respectively. The compound **3** showed no antimicrobial activity (*C* > 50 *μ*g/mL). In the ABTS cation radical scavenging assay, the inhibition rate of **3 **was 82.0% at 1 mg/mL (Positive control vitamin C, 75.2%, *C* = 0.1 mg/mL). The superoxide anion radical scavenging assay suggested that **3** showed 53.5% superoxide anion radical scavenging capacity at 2 mg/mL. While the positive control luteolin was 53.8% at 0.625 mg/mL. Unfortunately, the low available amount of compounds **1**, **2**, and **4 **precluded the antimicrobial and antioxidant assays.

## 3. Experimental

### 3.1. General Information

Column chromatography (CC): silica gel (SiO_2_, 200–300 mesh, Qingdao Marine Chemical Plant, Qingdao, P. R. China), MCI gel (Mitsubishi Chemical Corporation, Tokyo, Japan), and Sephadex LH-20 (GE Healthcare Bio-Sciences AB, USA). TLC: silica gel *GF*_254_ (Qingdao Marine Chemical Plant, Qingdao, P. R. China). UV absorbance of antioxide mixtures: Varioskan Flash Reader (ThermoFisher Scientific Inc., Waltham, MA). UV spectra: Perkin-Elmer S2 Lambda 35 UV/VIS spectrometer, λ in nm. IR spectra: Perkin-Elmer Spectrum One FT-IR spectrometer, as KBr tablets, ν in cm^−1^. Optical rotations: Perkin-Elmer 341 polarimeter. NMR spectra: Bruker Avance 600 MHz instrument, *δ* in ppm, *J* in Hz, residual solvent peak as reference. HR-ESI-MS: BioTOF-Q mass spectrometer, in *m/z.*

### 3.2. Material

The duckweed *Landoltia punctata* (G. Meyer) Les & Crawford was grown under natural conditions and collected in Kunming, Yunnan province, P. R. China. The duckweed was collected in May, washed with water, and then dried at 60 °C. The test strains *Aspergillus niger*, *Saccharomyces cerevisiae*, *Escherichia coli*, and *Bacillus subtilis* were obtained from Chengdu Institute of Biology, Chinese Academy of Sciences (CAS), P. R. China.

### 3.3. Extraction and Isolation

The powder (7 kg) of *L. punctata* was macerated in 95% ethanol (70 L) at room temperature for 4–5 days, twice. The extract was combined and concentrated under reduced pressure. Then the concentrate was suspended in hot water, and extracted with petroleum ether (P.E.), EtOAc, and *n*-BuOH successively. The P.E. extract was separated by silica gel column chromatography (CC) using P.E./acetone (10:1–1:1, *v/v*) as eluent, affording 4 fractions (Fr. 1-4), Fr. 2 and 3 were purified by Sephadex LH-20 CC to yield compounds **15**–**17** respectively. 

The EtOAc fraction (25 g) was subjected to a MCI column, and eluted with a stepwise gradient of EtOH/H_2_O (0:100, 20:80, 40:60, 60:40, 100:0, *v/v*) to give three fractions (I-III), which were further purified by HPLC (ODS-C18 column, 10 × 250 mm, flow rate 2.5 mL/min). Fr. I was separated using MeOH/H_2_O (40:60, containing 0.5% acetic acid, *v/v*) as the eluent to yield compounds **11** and **12**. Fr. II was purified using MeOH-H_2_O (45:55, containing 0.5% acetic acid, *v/v*) as the eluent to yield compounds **7**–**10**. Fr. III was separated using MeOH-H_2_O (60:40, containing 0.5% acetic acid, *v/v*) as the eluent to obtain compounds **5** and **6**. 

The same CC of the n-BuOH extract (60 g) on MCI gel as that of EtOAc extract gave four fractions (I–IV). Fraction II was subjected to preparative thin layer chromatography (TLC) and Sephadex LH-20 CC to afford compounds **1** (13 mg) and **2** (10 mg). Faction III was separated over silica gel with CHCl_3_/MeOH (7:1–0:1, *v/v*) as eluent to generate 4 fractions (IIIa-IIId). Fr. IIIb was separated by HPLC using MeOH/H_2_O (45:55, *v/v*) as the eluent to yield compounds **13** and **14**. Fr. IIIc and IIId were further purified by TLC and then Sephadex LH-20 CC to yield compounds **3** (18 mg) and **4** (8 mg) respectively. 

### 3.4. Hydrolysis

Acid hydrolysis. Compounds **1** and **2** (10 mg) were independently dissolved in aqueous solution of 2 N HCl (6 mL) and stirred at 85 °C overnight. After cooled down, the reaction mixture was neutralized and then extracted with CHCl_3_. The water layer were concentrated to dryness, dissolved in water to constant volume, and analyzed by comparing their TLC profile and optical rotation values with the standard sample of d-glucose [21,22], respectively.

Enzymatic hydrolysis. Compounds **3** and **4** (5.0 mg) were independently suspended in water (5 mL), and excessive *β*-d-glucosidase (Shanghai Zurui Biological Technology Co., Ltd., Shanghai, China) was added, respectively. The mixture was placed in water bath at 37 °C for 48 h. The products were analyzed by TLC [21].

### 3.5. Spectral Data

*(3β,24S)-9,19-Cycloartane-3,22,24,25-tetraol 3-O-[β-d-glucopyranosyl-(1→2)]-[β-*d*-glucopyranos-yl(1→6)]-β-d-glucopyranoside* (**1**): Amorphous powder. 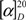 −3.0 (*c* 0.1, MeOH). IR (KBr) ν_max_ 3411, 2932, 1075 cm^−1^. ^1^H and ^13^C-NMR: See Table 1. HR-ESI-MS (positive mode) *m/z* 985.5371 [M+Na]^+^ (calc. for C_48_H_82_O_19_Na, 985.5343).

*(3β,24S)-9,19-Cycloartane-3,24,25-triol 3-O-[β-*d*-glucopyranosyl-(1→2)]-[β-d-glucopyranosyl-(1→6)]-β-d-glucopyranoside* (**2**): Amorphous powder. 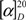 −4.0 (*c* 0.1, MeOH). IR (KBr) ν_max_ 3418, 2891, 1043 cm^−1^. ^1^H and ^13^C-NMR: See Table 1. HR-ESI-MS (positive mode) *m/z* 969.5406 [*M* + Na]^+^ (calc. for C_48_H_82_O_18_Na, 969.5393).

*3,4'-Dihydroxy-7,3'-dimethoxyflavan-5-O-β-d-glucopyranoside* (**3**): Colorless needles. 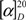 −42.0 (*c* 0.1, MeOH). UV (MeOH) λ_max_ (log ε) 218 (4.60), 227 (3.96), 282 (3.52) nm. IR (KBr) ν_max_ 3434, 1623, 1080 cm^−1^. ^1^H and ^13^C-NMR: See Table 2. HR-ESI-MS (negative mode) *m/z* 479.1549 (calc. for C_23_H_27_O_11_, 479.1559). 

*3*,*4'*-*Dihydroxy*-*4*,*7*,*3'*-*trimethoxyflavan*-*5*-*O*-*β*-*d*-*glucopyranoside* (**4**): Amorphous powder. 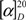 −20.0 (*c* 0.1, MeOH). UV (MeOH) λ_max_ (log ε) 226 (4.02), 280 (3.60) nm. IR (KBr) ν_max_ 3420, 2926, 1597, 1424, 1079 cm^−1^. ^1^H and ^13^C-NMR: See Table 2. HR-ESI-MS (negative mode) *m/z* 509.1662 (calc. for C_24_H_29_O_12_, 509.1664).

### 3.6. Biological Activity Assay

According to the reported [32], antifungal and antibacterial activities were determined using the Oxford cup method with Methicillin sodium as the positive control. The antioxidant activity assay was performed on the DPPH, ABTS^+•^, and superoxide anion radical scavenging models complying with the previously published literature [33].

**Table 1:** ^1^H (600 MHz) and ^13^C (150 MHz) NMR data of compounds **1** (in CD_3_OD, *J *in Hz) and **2 **(in C_5_D_5_N, *J* in Hz).

No.	1		2		No.	1		2	
	δ_C_	δ_H_	δ_C_	δ_H_		δ_C_	δ_H_	δ_C_	δ_H_
1	33.3	1.65–1.67 (*m*), 1.32–1.34 (*m*) (m)	30.3	2.50–2.53 (*m*), 1.25–1.26 (*m*)	25	74.0		73.1	
2	30.7	2.08–2.11 (*m*), 1.35–1.36 (*m*)	29.3	1.81–1.82 (*m*), 1.95–1.97 (*m*)	26	26.1	1.24 (*s*, 3H)	26.5	1.56 (*s*, 3H)
3	90.9	3.34–3.36 (*m*)	89.1	3.45–3.48 (*m*)	27	25.6	1.23 (*s*, 3H)	26.2	1.54 (*s*, 3H)
4	42.4		41.7		28	26.0	1.13 (*s*, 3H)	26.0	1.29 (*s*, 3H)
5	50.4	1.68–1.70 (*m*)	47.8	1.20–1.22 (*m*)	29	15.6	0.95 (*s*, 3H)	15.7	1.19 (*s*, 3H)
6	22.3	1.67–1.68 (*m*), 1.68–1.70 (*m*)	27.2	1.87–1.88 (*m*), 1.37 (overlapped)	30	20.1	1.01 (*s*, 3H)	18.6	0.99 (*s*, 3H)
7	27.4	1.39–1.40 (*m*), 1.36–1.37 (*m*)	33.5	1.50–1.51 (*m*), 1.53 (overlapped)	1'	105.3	4.52 (*d*, *J* = 7.20, 1H)	105.2	4.90 (*d*, *J *= 7.56, 1H)
8	49.7	1.60–1.63 (*m*)	53.3	1.64–1.65 (*m*)	2'	81.4	3.62–3.63 (*m*)	83.5	4.15–4.17 (*m*)
9	21.4		20.2		3'	78.2	3.61–3.62 (*m*)	77.3	4.10–4.11 (*m*)
10	27.5		26.9		4'	71.7	3.39–3.40 (*m*)	72.0	4.20–4.21 (*m*)
11	27.7	1.70–1.72 (*m*), 2.10–2.11 (*m*)	21.5	1.45–1.46 (*m*), 0.70–0.72 (*m*)	5'	77.1	3.52–3.54 (*m*)	77.2	4.03–4.04 (*m*)
12	34.2	1.72, 1.76, overlapped	32.6	1.64–1.65 (*m*), 1.24 (overlapped)	6'	70.2	3.85–3.87 (*m*), 4.16–4.18 (br. *d*)	70.4	4.29–4.31 (*m*), 4.82 (br. *d*)
13	46.9		45.9		1''	104.8	4.74 (*d*, *J *= 7.68, 1H)	106.3	5.35 (*d*, *J* = 7.62, 1H)
14	49.8		49.3		2''	76.5	3.28–3.29 (*m*)	75.6	4.04–4.05 (*m*)
15	37.0	1.40–1.41 (*m*), 1.42–1.43 (*m*)	36.7	1.65–1.66 (*m*), 1.55 (overlapped)	3''	78.4	3.60–3.61 (*m*)	78.3	4.24–4.25 (*m*)
16	28.4	1.42–1.43 (*m*), 2.03–2.05 (*m*)	28.8	1.37–1.38 (*m*), 1.63–1.64 (*m*)	4''	72.0	3.26–3,27 (*m*)	71.8	4.28–4.29 (*m*)
17	50.0	1.79–1.81 (*m*)	48.4	1.43–1.44 (*m*)	5''	78.1	3.30–3.31 (*m*)	78.6	3.90–3.92 (*m*)
18	18.7	1.11 (*s*, 3H)	19.9	0.84 (*s*, 3H)	6''	63.0	3.71–3.72 (*m*), 3.93–3.94 (*m*)	63.0	4.45–4.46 (*m*), 4.52–4.53 (*m*)
19	30.9	0.44 (*d*, *J* = 3.72)	30.0	0.24 (*d*, *J* = 4.08)	1'''	105.0	4.48 (*d*, *J* = 7.74, 1H)	105.7	5.13 ( *d*, *J* = 7.80, 1H)
		0.64 (*d*, *J* = 3.72)		0.48 (*d*, *J* = 4.08)	2'''	75.4	3.24–3.26 (*m*)	75.5	4.05–4.06 (*m*)
20	44.0	1.78–1.79 (*m*)	36.1	1.23–1.24 (*m*)	3'''	78.0	3.40–3.42 (*m*)	78.8	3.94–3.96 (*m*)
21	12.7	0.95 (*s*, 3H)	18.9	1.01 (*s*, 3H)	4'''	71.9	3.31–3.32 (*m*)	71.1	4.03–4.04 (*m*)
22	71.3	4.01 (br. *d*)	23.2	0.86–0.87 (*m*), 1.25 (overlapped)	5'''	78.1	3.29–3.30 (*m*)	78.4	4.21–4.22 (*m*)
23	32.9	1.40–1.41 (*m*), 1.53–1.57 (*m*)	34.5	1.83–1.85 (*m*), 1.68 (overlapped)	6'''	63.3	3.70–3.71 (*m*), 3.90–3.92 (*m*)	63.1	4.33–4.36 (*m*), 4.48–4.50 (*m*)
24	76.2	3.58–3.60 (*m*)	79.4	3.78–3.81 (*m*)					

**Table 2:** ^1^H (600 MHz) and ^13^C (150 MHz) NMR data of compounds **3** and **4** (in CD_3_OD, *J* in Hz).

No.	3		4	
	δ_C_	δ_H_	δ_C_	δ_H_
2	80.3	4.98 (br. *s*, 1H)	78.6	5.05 (br. *s*, 1H)
3	67.4	4.29 (br. *s*, 1H)	69.2	4.04 (br. *d*, 1H)
4	29.7	3.09 (*d*, *J* = 17.60, 1H)3.02(1H, dd)	73.5	4.62 (*d*, *J* = 2.28, 1H)
		3.02 (*dd*, *J* = 17.60, 4.38, 1H)		
5	158.6		157.9	
6	97.1	6.49(*d*, *J* = 2.10)	96.6	6.54 (*d*, *J* = 1.80)
7	160.9		162.9	
8	96.5	6.29 (*d*, *J* = 2.10)	96.3	6.30 (*d*, *J* = 1.80)
9	157.3		160.4	
10	104.0		104.4	
1'	132.3		131.6	
2'	112.1	7.22 (*d*, *J* = 1.06)	112.3	7.22 (*d*, *J* = 1.10)
3'	148.8		149.0	
4'	147.2		147.4	
5'	115.9	6.87 (*d*, *J* = 8.16)	116.0	6.90 (*d*, *J* = 8.04)
6'	120.8	6.99 (*dd*, *J* = 1.06, 8.16)	121.0	7.00 (*dd*, *J* = 1.10, 8.04)
1''	102.8	4.95 (*d*, *J* = 7.20)	102.6	4.99 (*d*, *J* = 7.50)
2''	75.1	3.50–3.55 (overlapped)	75.3	3.58–3.59 (overlapped)
3''	78.4	3.50–3.55 (overlapped)	78.5	3.53–3.56 (overlapped)
4''	71.6	3.47–3.48 (*m*)	71.6	3.46–3.48 (overlapped)
5''	78.2	3.50–3.55 (overlapped)	76.9	3.53–3.56 (overlapped)
6''	62.7	3.79 (*dd*, *J *= 5.58, 12.10)	62.8	3.79 (*dd*, *J* = 5.00, 12.20)
		3.98 (*d*, *J* = 12.10)		4.00 (*d*, *J* = 12.20)
MeO-4			56.6	3.65 (*s*, 3H)
MeO-7	56.6	3.82 (*s*, 3H)	56.0	3.84 (*s*, 3H)
MeO-3'	56.0	3.95 (*s*, 3H )	57.8	3.96 (*s*, 3H)

## 4. Conclusions

In this study, we identified 12 flavonoids including two new flavanol glucosides from *L. punctata*. The result showed that apigenin or luteolin flavonoids are the main constituents of this species, which is in good agreement with the previous reports and our previous study results that the transcripts for key enzymes of flavonoid biosynthesis in *L. punctata* expressed in high abundance at the transcriptional level [9,10,34]. Flavonoids are generally biosynthesized to cope with environmental stressors such as ultraviolet radiation, ozone, heavy metals, nutrient limitation, herbivores, and so on. The high content of flavonoids in *L.* *punctata* could be related to environmental stressors. Meanwhile cycloartane triterpenoids were discovered in Lemnaceae family for the first time in this study. Many cycloartane triterpenoids possessed diverse bioactivities such as anti-inflammatory, anti-tumor, anti-viral, immuno-regulatory, hypoglycemic, cardiovascular system, nervous system and hepato-protective effects [35,36], some of which offer good prospectives in medical applications. This study demonstrates that *L.* *punctata* is a new source for cycloartane triterpenoids. 
